# Prevention of human milk-acquired cytomegalovirus infection in very-low-birth-weight infants

**DOI:** 10.1186/s12887-023-04044-8

**Published:** 2023-05-18

**Authors:** Mi Lim Chung, Heungsup Sung, Euiseok Jung, Byong Sop Lee, Ki Soo Kim, Ellen Ai-Rhan Kim

**Affiliations:** 1 Department of Pediatrics, University of Inje College of Medicine, Haeundae Paik Hospital, Busan, South Korea; 2 Department of Laboratory Medicine, University of Ulsan College of Medicine, Asan Medical Center, Seoul, South Korea; 3 Department of Pediatrics, University of Ulsan College of Medicine, Asan Medical Center Children’s Hospital, Seoul, South Korea

**Keywords:** Cytomegalovirus, Human milk, Very low birth weight infant, Pasteurization

## Abstract

**Background:**

The aim of the study was to determine the rate of cytomegalovirus virolactia in the human milk (HM) of mothers of VLBW infants, compare the CMV infection rates and the changes in CMV DNA viral load and nutrient profile among different HM preparation methods.

**Methods:**

A prospective randomized controlled study was performed in infants with gestational age < 32 weeks or birth-weight < 1500 g admitted to neonatal intensive care unit of Asan Medical Center and Haeundae Paik Hospital who were given mother’s own milk. Enrolled infants were randomized into three groups according to the HM preparation methods: freezing-thawing (FT), FT + low-temperature Holder pasteurization (FT + LP), and FT + high-temperature short-term pasteurization (FT + HP). Urine CMV culture and PCR were obtained at birth and at 4, 8, and 12 weeks. HM CMV culture and PCR were obtained at birth and at 3, 6, 9, and 12 weeks. Changes in macronutrients in HM was obtained at 4 ~ 6 weeks.

**Results:**

Of 564 infants, 217 mothers (38.5%) produced CMV PCR positive milk. After exclusion, a total of 125 infants were randomized into the FT (n = 41), FT + LP (n = 42), and FT + HP (n = 42) groups, whose rate of HM-acquired CMV infection was 4.9% (n = 2), 9.5% (n = 4), and 2.4% (n = 1), respectively. Out of seven CMV infected infants, two infants fed with FT + LP HM developed CMV infection- associated symptoms. Ages at diagnoses were earlier (28.5 days after birth) and at younger post conceptional age (< 32 weeks) in comparison to infants with asymptomatic CMV infection. CMV DNA viral load significantly decreased after pasturizations, especially in FT + HP group.

**Conclusions:**

HM-acquired symptomatic CMV infection rate is low and its impact on clinical course was not serious in our VLBW infants. However, evidences showing poor neurodevelopmental outcome in later life, we need to generate a guideline to protect VLBW infant form HM transmitted CMV infection. Based on our small sized study, we did not find any superiority in pasteurizing HM with frequently used LP in comparison to frozen or HP HM. More research is needed to determine the method and duration of pasteurization to reduce the HM-acquired CMV infection.

**Supplementary Information:**

The online version contains supplementary material available at 10.1186/s12887-023-04044-8.

## Introduction

Localized reactivation of cytomegalovirus (CMV) in breasts during lactation may occur in CMV immunoglobulin (Ig) G-seropositive women, and reactivated CMV is excreted via human milk (HM) [1–3]. Postnatal HM-acquired CMV infection is rare and usually asymptomatic in full-term neonates, likely due to the protective effect of maternal antibodies [4]. However, preterm infants may be at risk of symptomatic postnatal CMV infection because of the relative lack of maternal antibodies [5–7].

HM is the primary route of CMV infection in very-low-birth-weight (VLBW) infants since the introduction of CMV-negative, leukocyte-free blood products reduced transfusion-related CMV infection [8]. However, the advantages of HM intake among preterm infants may outweigh the risk of CMV infection, and it is therefore advised not to withhold the mother’s own HM even though the mother is seropositive for CMV IgG [9].

Numerous efforts have been made to find an effective method for decreasing the risk of CMV transmission via HM. This is a particularly important issue in South Korea, where the seropositive rate for CMV is over 95% among reproductive-aged mothers [10, 11] We therefore conducted a randomized controlled study to determine the rate of CMV virolactia among mothers providing HM for their VLBW infants and compared the CMV infection rates among three different HM preparation methods. Additionally, we analyzed the changes in CMV DNA load and nutrients in HM after treatment.

## Methods

This prospective randomized double blind study was carried out at the neonatal intensive care units (NICUs) of Asan Medical Center (Seoul, South Korea) and Haeundae Paik Hospital (Busan, South Korea) after obtaining approval by the respective institutional review boards (Asan Medical Center, #2015 − 0367; Haeundae Paik Hospital, #129792-2015-039). All subjects have given their written informed consent.

### Subjects & sub-grouping

Neonates born with a gestational age (GA) < 32 weeks or birth weight (BW) < 1500 g between April 2015 and January 2019 and whose mother’s HM showed positive results of CMV in culture or polymerase chain reaction (PCR) within 7 days after birth were enrolled. The exclusion criteria were congenital CMV infection, death within four weeks of age, critical illness, transfer out, unavailable HM supply by the mother. The enrolled infants were randomized into three subgroups according to the HM preparation methods: the freeze-thawing (FT) group, FT + low-temperature Holder pasteurization (FT + LP) group, and the FT + high-temperature short-term pasteurization (FT + HP) group. Specifically, infants in the FT group were fed their mother’s own HM that had been stored at -20 °C for more than three days. Infants in the FT + LP group were fed mother’s own HM that had been conventionally pasteurized at 62.5 °C for 30 min after thawing. Infants in the FT + HP group were fed HM that had been pasteurized at 72 °C for 5 s after thawing. After heating in a shaking water bath (BS-11, Jeio Tech, Korea), the HM was directly immersed in iced water for 15 min until the temperature reached 10.0 °C, and the HM was stored in a refrigerator; the heating treatment was carried out by one designated research nurse at each hospital using the same protocol. The randomization was performed at the Department of Clinical Epidemiology and Biostatistics at Asan Medical Center.

### Laboratory tests

CMV culture and PCR in urine and HM: CMV culture and PCR were checked in urine samples within 7 days and at 4, 8, and 12 weeks. CMV culture and PCR in HM samples in each group after preparation were checked within 7 days and at 3, 6, 9, and 12 weeks.

Quantification of CMV PCR: Paired samples of fresh-frozen, frozen-low temperature pasturized HM, frozen-high temperature pasturized HM were sent for CMV PCR quantitative analysis. CMV DNA load (copies/mL) was measured using quantitative PCR assay. CMV DNA extraction was performed using commercially available QIAsymphony Virus/Bacer Kits (QIAGEN, Hilden, Germany). CMV PCR quantitative test was performed using the Artus® R CMV PCR test (QIAGEN) in Rotor-gen (QIAGEN).

CMV glycoprotein B (gB) PCR for genotyping: Genetic analysis of viral mutations was performed in CMV-infected infants. When entering the CMV strain, the gB gene was amplified using the following initiator: 5’’-CAA GAR GTG AAC ATG TCC GA-3’, 5’’-GTC ACG CAG CTG GCC AG-3’. Primary PCR amplification products were double-PCR (nested PCR) with 5′′-TGG AAC TGG AAC GTT TGG C-3′, 5′′-GAA ACG CGC GGC AAT CGG-3′. The PCR conditions were described by Binder et al. [12] Sequence analysis of the amplified products was performed by Macrogen (Seoul, South Korea).

CMV strain typing: The CMV gB genotype was sorted by the ClustalW program using the AD 169 strain (GenBank Accession No. FJ527563), Toledo strain (GU937742), Towne strain (FJ616285), and MEGA 7 (Arizona State University, Arizona, USA). Phylogenetic analysis was performed by the joining method.

Macronutrient changes in HM: Nutritional components in HM in each group after preparations were measured using the Miris® Human Milk Analyzer (Miris, Uppsala, Sweden).

### Statistical analysis

Differences among groups were assessed using the chi-squared test or Fisher’s exact test for categorical variables and the independent *t*-test or Mann–Whitney *U* test for continuous variables. Analysis of variance (ANOVA) with Scheffe’s post-hoc test or Kruskal–Wallis test with Dunn’s post-hoc test was also employed. The Shapiro–Wilk test was used to determine if a continuous variable had a normal distribution. Linear mixed model was used for taking into account the data for the same subject up to four times is considered. Univariate and multivariate analyses were performed using linear and logistic regression analyses. The receiver operating characteristic curve was used to assess the sensitivity and specificity of the CMV titer for predicting infection. All statistical analyses were carried out using SPSS 24.0 and statistical significance was set *p-value < 0.05*.

## Results

### Basic demographic and clinical characteristics

**Fig. 1:**
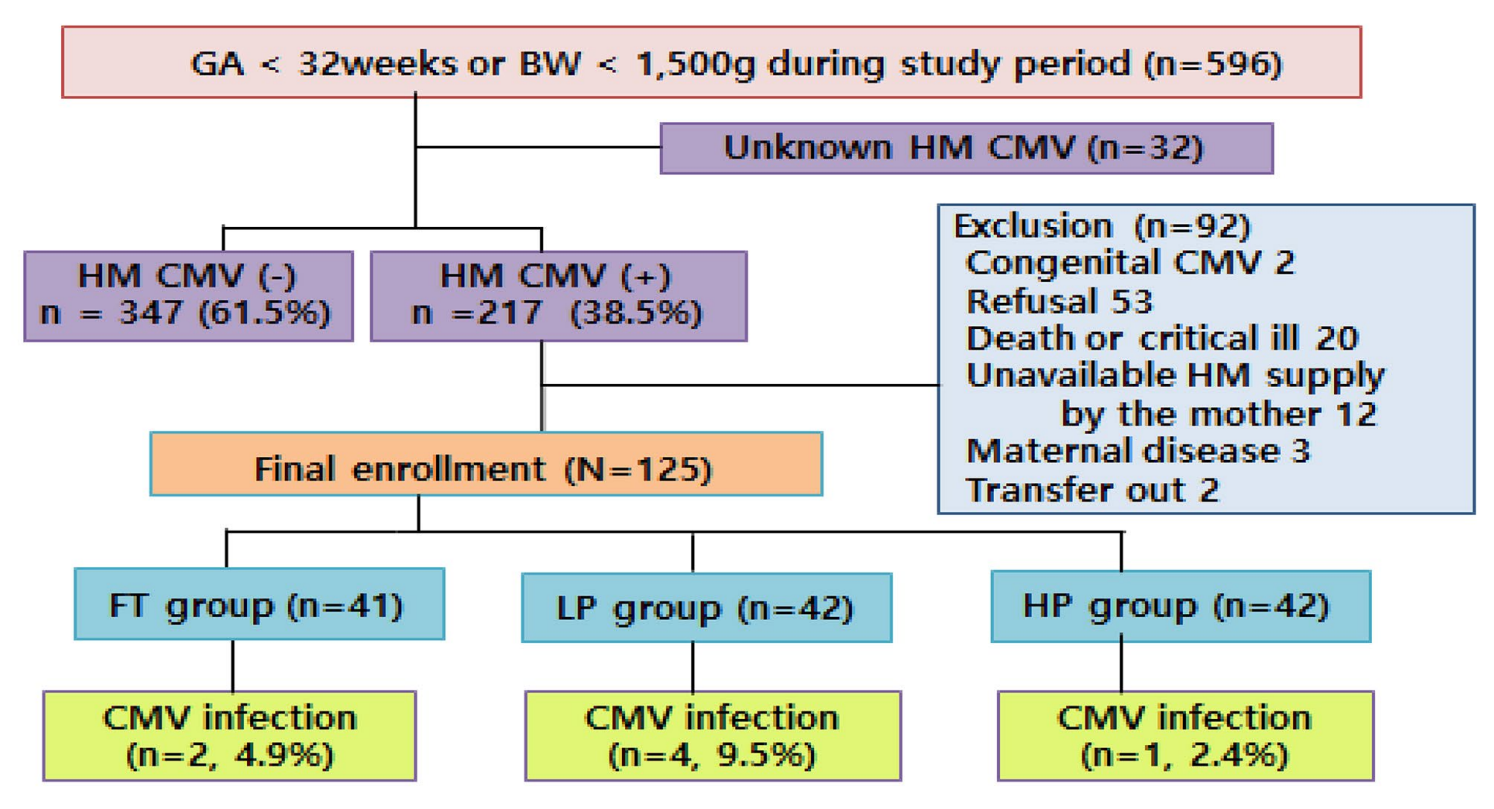
Study flow sheet

During the study period, 596 neonates with a GA less than 32 weeks or BW less than 1500 g were admitted. Of them, 564 neonates were tested for HM CMV and 217 (38.5%) showed a positive result. After excluding 92 infants, a total of 125 infants were enrolled in the study and randomized into the FT (n = 41), FT + LP (n = 42), and FT + HP (n = 42) groups (Fig. 1), whose rate of CMV PCR positivity was 4.9%, 9.5%, and 2.4%, respectively (*p-value 0.40*). There were no significant demographic and clinical characteristic differences among the three groups (Table 1).

**Table 1:** Comparison of basic demographic and clinical characteristics between three groups

Variables	FT (n = 41)	FT + LP (n = 42)	FT + HP (n = 42)	*P -value*
**Gestational age (weeks)**	28.32 ± 2.88	27.66 ± 2.91	27.81 ± 2.46	*0.82* ^*1*^
**Body weight (g)**	1090.85 ± 399.33	1017.90 ± 368.30	1020.83 ± 288.04	*0.57* ^*3*^
**Delivery type (NSVD / CS)**	9 / 32	13 / 29	7 / 35	*0.29* ^*1*^
**Sex (male / female)**	19 / 22	20 / 22	24 / 18	*0.56* ^*1*^
**Small for gestational age**	13 (31.7)	9 (21.4)	11 (26.2)	*0.58* ^*3*^
**Surfactant use**	31 (75.6)	35 (83.3)	36 (85.7)	*0.46* ^*1*^
**Bronchopulmonary dysplasia**	21 (51.2)	25 (59.5)	28 (66.7)	*0.36* ^*1*^
Significant patent ductus arteriosus*	18 (43.9)	20 (47.6)	16 (38.1)	*0.67* ^*1*^
**Retinopathy of prematurity (≥ stage 2)**	7 (17.1)	8 (19.0)	11 (26.2)	*0.56* ^*1*^
**Necrotizing enterocolitis**	3 (7.3)	5 (11.9)	9 (21.4)	*0.16* ^*1*^
**Sepsis**	12 (29.3)	17 (40.5)	18 (42.9)	*0.40* ^*1*^
**Total parental nutrition days**	34.37 ± 23.04	41.31 ± 25.44	47.52 ± 35.31	*0.20* ^*4*^
**Days to achieve full enteral feeding**	37.66 ± 33.04	43.02 ± 29.67	44.31 ± 31.49	*0.30* ^*4*^
**Average HM intake (mL/day)**	86.94 ± 62.65	82.15 ± 59.78	75.43 ± 50.04	*0.93* ^*4*^
**Proportion of HM in total enteral intake**				
< 25%	4 (9.8)	5 (11.9)	7 (16.7)	*0.76* ^*1*^
25 – 49%	17 (41.5)	12 (28.6)	14 (33.3)	
50 – 75%	11 (26.8)	11 (26.2)	12 (28.6)	
> 75%	9 (22.0)	14 (33.3)	9 (21.4)	
**Duration of HM feeding (days)**	40.46 ± 17.91	41.29 ± 18.61	42.46 ± 20.03	*0.58* ^*4*^
**Numbers of transfusion**				
Packed red blood cell	6.07 ± 8.60	9.33 ± 12.00	6.81 ± 7.61	*0.67* ^*4*^
Platelet concentrate	2.22 ± 4.71	6.38 ± 14.36	2.55 ± 5.49	*0.56* ^*4*^
Fresh frozen plasma	1.10 ± 3.40	2.67 ± 12.30	0.62 ± 1.06	*0.67* ^*4*^
**CMV infection**	2 (4.9)	4 (9.5)	1 (2.4)	*0.40* ^*2*^
**Outcome (survival / death)**	40 / 1	39 / 3	41 / 1	*0.41* ^*1*^

Values are either number (%) or mean ± standard deviation

*Defined as conditions needing medical or surgical treatment

^1^Chi-squared test, ^2^Fisher’s exact test, ^3^ANOVA, ^4^Kruskal–Wallis test

**Abbreviations:** FT, freeze-thawing; LP, low-temperature Holder pasteurization; HP, high-temperature short-term pasteurization; NSVD, normal spontaneous vaginal delivery; CS, cesarean section; HM, human milk

### Comparison of infants according to CMV infection

Seven (5.6%) infants were diagnosed with CMV infection at an average of 44 days after birth. When the infants were compared according to the presence of CMV infection, there were no significant differences except for the higher prevalence of single pregnancy among the CMV-infected infants (Table 2).

**Table 2:** Comparison according to CMV infection

Variables	CMV infection					Univariate logistic regression analysis		
	Yes (n = 7)		No (n = 118)	*P-value*		Odds ratio (95% CI)		*P-value*
**Gestational age (weeks)**	27.86 ± 3.44		27.73 ± 2.68	*0.63* ^*2*^		0.97 (0.71–1.31)		*0.81*
**Body weight (g)**	1012.86 ± 271		1044.59 ± 358	*0.82* ^*2*^		1.00 (1.00–1.00)		*0.82*
**Delivery type (NSVD / CS)**	3 / 4		26 / 92	*0.35* ^*1*^		0.38 (0.08–1.79)		*0.22*
**Sex (male / female)**	2 / 5		61 / 57	*0.27* ^*1*^		2.68 (0.50-14.34)		*0.25*
**Small for gestational age**	1 (14.3)		20 (16.9)	*1.00* ^*1*^		0.79 (0.14–4.56)		*0.80*
**Type of pregnancy**								
**Single / twin or triplet**	6 / 1		47 / 71	*0.04* ^*1*^		0.11 (0.01–0.95)		*0.04*
**Surfactant use**	6 (85.7)		96 (81.4)	*1.00* ^*1*^		1.37 (0.16–12.01)		*0.77*
**Bronchopulmonary dysplasia**	4 (57.1)		70 (59.3)	*1.00* ^*1*^		0.91 (0.20–4.27)		*0.91*
Significant patent ductus arteriosus*	3 (42.9)		51 (43.2)	*1.00* ^*1*^		0.99 (0.21–4.60)		*0.98*
**ROP (≥ stage 2)**	2 (28.6)		24 (20.3)	*0.63* ^*1*^		1.57 (0.29–8.58)		*0.60*
**Necrotizing enterocolitis**	0 (0.0)		17 (14.4)	*0.59* ^*1*^		N/E		
**Sepsis**	2 (28.6)		45 (38.1)	*0.71* ^*1*^		0.65 (0.12–3.49)		*0.61*
**Numbers of transfusion**								
Packed red blood cell	11.43 ± 11.47		7.18 ± 9.50		*0.38* ^*1*^	1.04 (0.97–1.11)		*0.27*
Platelet concentrate	5.29 ± 9.39		3.64 ± 9.46		*0.97* ^*1*^	1.01 (0.95–1.08)		*0.66*
Fresh frozen plasma	0.43 ± 0.79		1.53 ± 7.62		*0.74* ^*1*^	0.83 (0.39–1.76)		*0.62*
**Total parental nutrition days**	47.57 ± 28.59		40.74 ± 28.83		*0.42* ^*3*^	1.01 (0.98–1.03)		*0.54*
**Days to achieve full enteral feeding**	44.14 ± 31.68		41.55 ± 31.40		*0.69* ^*3*^	1.00 (0.98–1.03)		*0.83*
**Average HM intake (mL/day)**	80.55 ± 45.74		81.51 ± 58.21		*0.70* ^*3*^	1.00 (0.99–1.01)		*0.97*
**Proportion of HM in total enteral intake**								
< 25%		1 (14.3)	15 (12.7)	0.33^1^		Reference		
25% – 49%		1 (14.3)	42 (35.6)			0.36 (0.02–6.08)		*0.48*
50% – 75%		4 (57.1)	30 (25.4)			2.00 (0.21–19.50)		*0.55*
> 75%		1 (14.3)	31 (26.3)			0.48 (0.03–8.28)		*0.62*
**Study group**								
FT		2 (28.6)	39 (33.1)	*0.40* ^*1*^		Reference		
FT + LP		4 (57.1)	38 (32.2)			2.05 (0.35–11.87)		*0.42*
FT + HP		1 (14.3)	41 (34.7)			0.48 (0.04–5.46)		*0.55*

Values are either n (%) or mean ± standard deviation

*Defined as conditions needing medical or surgical treatment

^1^Fisher’s exact test, ^2^Independent t-test, ^3^Mann–Whitney *U* test, N/E: not estimable

**Abbreviations:** CMV, cytomegalovirus; CI, confidence interval; NSVD, normal spontaneous vaginal delivery; CS, cesarean section; ROP, retinopathy of prematurity; HM, human milk; FT, freeze-thawing; LP, low-temperature Holder pasteurization; HP, high-temperature short-term pasteurization

### Quantitative analysis of CMV DNA in HM

**Fig. 2:**
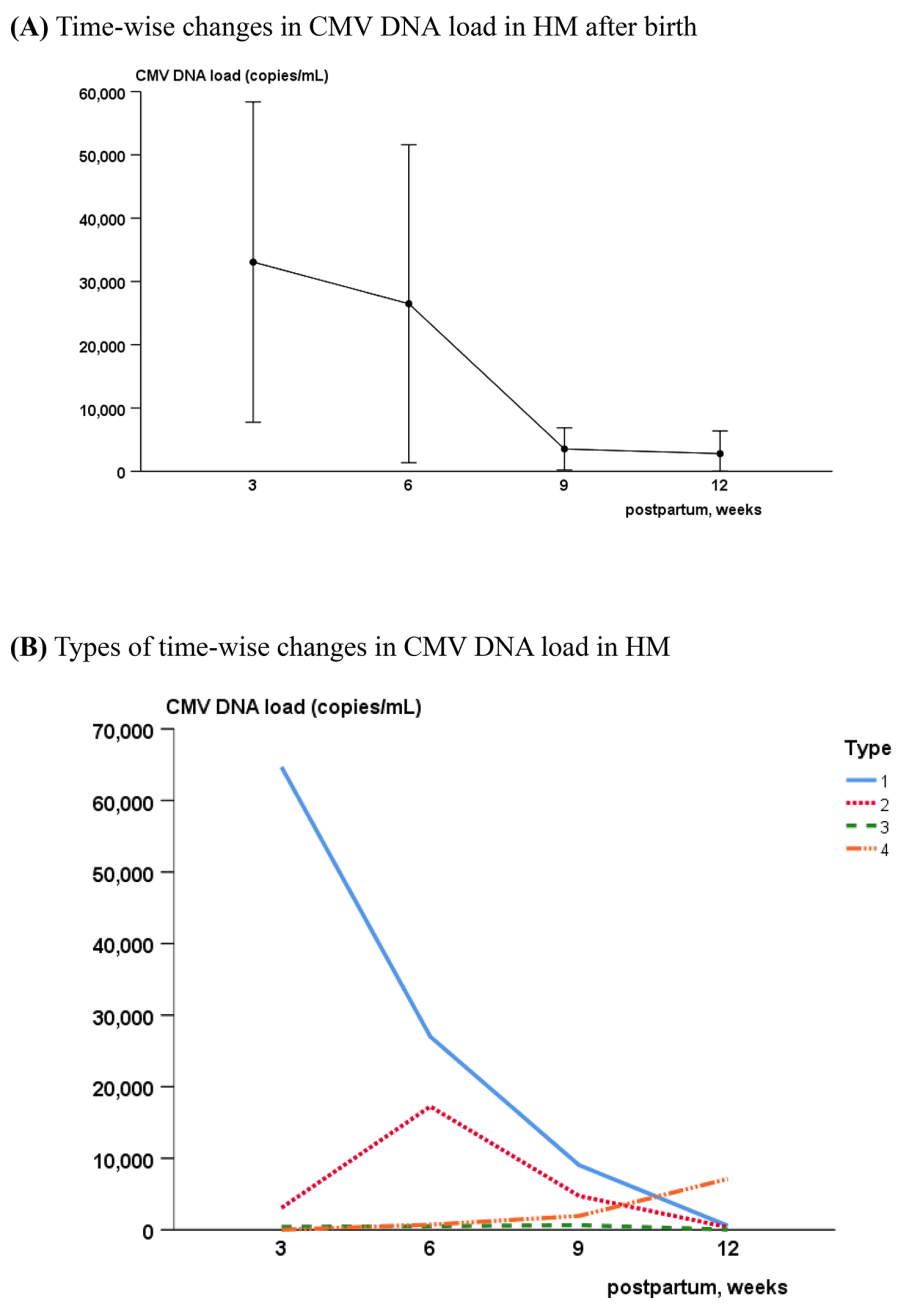
CMV DNA load in HM (Values are presented as mean ? standard deviation)

The mean value of CMV DNA load (copies/mL) was the highest at 3 weeks after birth and gradually decreased over 2–3 months (Fig. 2A). Specifically, the time-wise changes in CMV DNA load values could be classified into four types (Fig. 2B): gradual decrease after week 3 (43%; type 1), peak at week 6 and gradual decrease (18%; type 2), mild increase until week 9 and decrease (14%; type 3), and gradual increase until week 12 (12%; type 4). The remaining 14% of HM samples did not show a consistent pattern and could not be categorized.

The CMV DNA load in HM was decreased after preparation, especially FT + HP method and this decline was particularly evident in 9 and 12 weeks samples (Table 3, *p < 0.05*). However, CMV DNA load was not associated with the risk of CMV infection (data not shown).

**Table 3:** Changes of CMV DNA load in HM before and after treatment in each group In case of using all data

	FT		*p-*	FT + LP		*p-*	FT + HP		*p-*	*overall*
	Lsmean	SE	*value*	Lsmean	SE	*value*	Lsmean	SE	*value*	*p-value*
In case of using all data										
Fresh	2.354	0.314		2.871	0.312		2.565	0.283		*0.9782*
Post	2.287	0.314		2.681	0.312		2.122	0.283		
Difference	0.067	0.078	*0.400*	0.190	0.066	*0.01*	0.443	0.064	*< .0001*	*0.001*
In case of using only 9 and 12 weeks data										
Fresh	2.493	0.452		3.257	0.366		2.905	0.328		*0.7308*
Post	2.484	0.449		2.944	0.367		2.293	0.329		
Difference	0.010	0.098	*0.9225*	0.313	0.072	*0.0002*	0.612	0.071	*< .0001*	*0.0001*

**Abbreviations:** FT, freeze-thawing; LP, low-temperature Holder pasteurization; HP, high-temperature short-term pasteurization; Lsmean, least square mean; SE, standard error

**Table 4:** Clinical characteristics of the 7 infants with CMV infection

No.	GA (weeks), BW (gram)	Sex	Number of transfusions (PRBC / PC / FFP)	Study group	Age at diagnosis (PCA)	CMV-positive specimen	Average HM intake (mL/day)	Symptom	Duration of gancyclovir treatment	Outcome
1	25 + 2, 720	F	17 / 0 / 0	FT + LP	28 (29 + 2)	Blood, urine, BAL, CSF	71	Lung	3 weeks	Survived
2	28 + 6, 1210	M	6 / 0 / 0	FT + HP	28 (32 + 6)	Urine	89	None	2 weeks	Survived
3	27 + 2, 1040	F	25 / 23 / 0	FT + LP	29 (31 + 3)	Blood, urine, sputum	11	Lung, SLS (PLT↓, AST/ALT↑)	3 weeks	Died
4	26 + 6, 1080	F	5 / 0 / 6	FT	83 (39 + 2)	Urine	153	None	N/A	Survived
5	24 + 0, 630	M	27 / 14 / 2	FT + LP	56 (32 + 0)	Urine	104	None	N/A	Survived
6	33 + 4, 1420	F	0 / 0 / 0	FT + LP	28 (37 + 4)	Urine	41	None	N/A	Survived
7	32 + 6, 990	F	0 / 0 / 0	FT	56 (40 + 6)	Urine	94	None	N/A	Survived

**Abbreviations:** CMV, cytomegalovirus; GA, gestational age; BW, body weight; PCA, post-conceptional age; F, female; M, male; BAL, bronchoalveolar lavage; CSF, cerebrospinal fluid; SLS, sepsis-like symptoms; PLT, platelet; AST/ALT, aspartate aminotransferase/alanine aminotransferase; N/A, not available

### Analysis of CMV glycoprotein B genotype

**Fig. 3:**
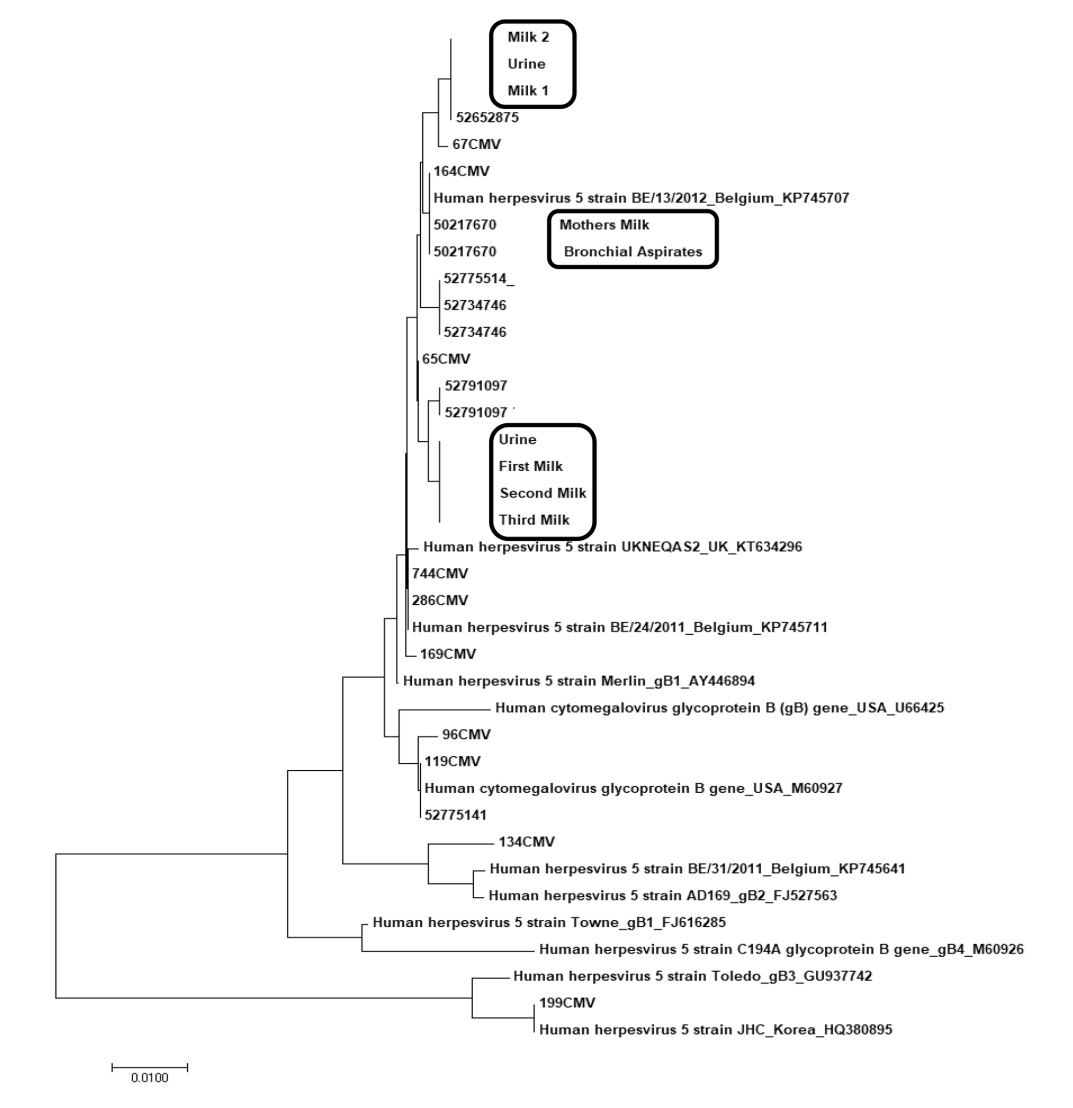
CMV glycoprotein B genotyping

CMV gB was confirmed to be the same in both urine samples and HM, and in one bronchoalveolar lavage fluid sample and HM (Fig. 3).

### Changes in macronutrients of HM after preparation

Changes in the nutrients of HM after preparation were analyzed in 79 infants. There was no change in HM after preparation, except for the significant decreases in energy in frozen HM than in HM prepared by other methods (data not shown).

### Clinical course of CMV-infected infants

CMV infection was diagnosed in seven infants. Two infants with CMV-associated symptoms were diagnosed at 28 and 29 days, and their post-conceptional age at diagnosis was 29 + 2 and 31 + 3 weeks, respectively (Table 4). Both symptomatic infants were in the FT + LP group.

## Discussion

In previous studies, the rates of HM-acquired CMV infection had a wide range from 10 to 40%, and the rate of symptomatic CMV infection also had a wide range from 1 to 10% [13–15]. In the present study, the rates of CMV infection rate and symptomatic infection were 5.6% and 1.6%, respectively. The large differences among reports are likely due to maternal and viral factors [16, 17]. In addition, Hamprecht et al. pointed out that the unexpectedly low incidence of HM-acquired CMV infection can be biased by using pre-treated HM [18] Although it is clear that pre-treatment (e.g., freezing, pasteurization) has an effect on the development of HM-acquired CMV infection, many studies have not critically discussed this. There are well-known risk factors for CMV infection such as younger GA and lower BW [6, 15], but such risk factors could not be identified in this study; this may be due to restricting the subjects to those with a GA less than 32 weeks and BW less than 1500 g.

Two out of seven (28.6%) children with CMV infection showed CMV-associated symptoms. Symptomatic CMV infection is more common in premature infants because of their immature immune system and relative lack of maternal IgG [18]. As such, two patients with symptoms were diagnosed at around 4 weeks of age, whereas five patients with asymptomatic infection were diagnosed between 4 and 12 weeks of age. Therefore, it seems that early infections tended to develop symptoms associated with HM-acquired CMV infection, which is consistent with the results of previous studies [17–19]. HM-acquired CMV infection is usually asymptomatic in full-term neonates or has a favorable prognosis, even in preterm infants [20–23]. However, some studies have reported adverse long-term prognosis of symptomatic HM-acquired CMV infection in very preterm infants [24, 25]. Therefore, efforts should be made to actively reduce the risk of symptomatic CMV infection acquired through HM.

Numerous efforts have been made to reduce the risk of HM-acquired CMV infection by pre-treating HM [26]. Lanzieri et al. conducted a meta-analysis of 17 studies and showed the effect of freezing HM on CMV infection and the development of CMV-related sepsis-like syndrome (CMV-SLS) [17] In the case of feeding frozen HM, the CMV infection rate was slightly low but there was no significant difference in CMV-SLS; hence, freezing alone is limited in preventing symptomatic CMV infection. Several other studies also reported that freezing HM at − 20 degrees reduced the risk of CMV infection but did not completely prevent it [27–29]. Heat sterilization is another pre-treatment method for HM, of which the Holder pasteurization method using heating at 63–65 ℃ for 30 min is traditionally used and the short-term high-temperature sterilization method has recently been studied. Holder pasteurization is the most effective method considering its safety against viral infection, but it has disadvantage of reducing the biological activities of various components in HM, such as growth factors, lysozyme, immunoglobulin, lactoferrin, enzymes, and some cytokines and vitamins [30] Short-term high-temperature sterilization (72 ℃ for 5 s) shows a similar effect in CMV DNA reduction as Holder pasteurization and is known to be excellent at preserving the activities of growth hormones and enzymes [31–33]. In addition, sterilization using microwave radiation at high power (750 W) [34] or 254 nm ultraviolet-C (UV-C) irradiation has also been studied [35]. The CMV DNA load in HM was decreased after preparation, especially FT + HP method and this decline was particularly evident in 9 and 12 weeks samples. However, CMV DNA load was not associated with the risk of CMV infection in this present study and it might be due to the small number of subjects. In addition, the FT + HP group had the lowest infection rate (1/42, 2.4%), but a statistically significant difference was not found among the groups. The lack of statistical difference is ultimately due to the same reason.

Since the CMV positivity rate in women of childbearing age varies greatly from country to country [11], it is necessary to provide region- and country-specific guidelines for breastfeeding to reduce the risk of CMV infection. In Germany, it is recommended that VLBW babies are fed sterile HM for 6 weeks [36]. The Austrian Academy of Pediatrics recommends that CMV seropositive nursing mothers add sterilization until 34 weeks after fertilization [37]. In France, sterilization is added until 32 weeks in preterm infants under 28 weeks of GA and under 1000 g of BW if the mother’s CMV antibody is positive or the result is unknown; otherwise, fresh HM is recommended [38]. On the other hand, it is also suggested that freezing alone may be sufficient because the occurrence of CMV infection by HM is very rare and long-term complications are not worrisome [39]. Although there are several guidelines like these, they are not absolute, and they seem to have different attitudes for each NICU, for each physicians.

Studies have shown that reactivated CMV begins to be secreted through HM within a few days after birth, even in colostrum, and its load gradually increases, peaking between 3 and 6 weeks after birth and then gradually decreasing over 2–3 months during lactation [18, 40], which is similar to the results of this study. However, the CMV DNA load in 14% of HM samples continued to increase even after 9 weeks, so it was not possible to confirm that CMV transmission had ended and that there is no risk of infection after 12 weeks of age. In addition, although the initial HM CMV result was negative, HM-acquired CMV infection can develop in the future. Likewise, the onset, dynamics, and termination of virus shedding into the HM vary. Therefore, in addition to accounting for country-specific circumstances, an individualized follow-up plan is necessary considering the fact of the variety of dynamics of the CMV in HM.

This study has some limitations. A correlation between CMV DNA load and infection or a statistically significant difference in infection rate according to sterilization method could not be confirmed, which is thought to be due to the small number of patients. Since the CMV infection rate is low, only 7 infants were infected. Moreover, CMV DNA titer values could not be measured in all patients or at all time points. Also, CMV DNA dose not fully refect the CMV infectivity. And several important factors including protective antiodies or immue status could not be considered. However, this study is the first randomized controlled double blind trial to investigate the association between postnatal HM-acquired CMV infection and pasteurization in premature infants.

## Conclusions

HM-acquired CMV infection is low and its impact on clinical course was not serious enough to discontinue HM feeding in our VLBW infants. However, evidences showing poor neurodevelopmental outcome in later life, we need to generate a guideline to protect VLBW infant form HM transmitted CMV infection. Based on our small sized study, we did not find any superiority in pasteurizing HM with frequently used LP in comparison to frozen or HP HM. More research is needed to determine the method and duration of pasteurization to reduce the HM-acquired CMV infection.

## Electronic supplementary material

Below is the link to the electronic supplementary material.

Supplementary Material 1

## Data Availability

This study was approved by two institutional IRB’s where study was done. However, parental consents nor they were informed for possible public availability of their children’s medical information. Such statements were not included as a part of the consent form. Therefore, the research data cannot be open to public. he underlying datasets are available from the corresponding author on reasonable request.

## Abbreviations

CMV: Cytomegalovirus
Ig: immunoglobulin, HM:human milk
VLBW: very low birth weight
NICU: neonatal intensive care unit
GA: gestational age
BW: birth weight
PCR: polymerase chain reaction
FT: freeze-thawing
LP: low-temperature Holder pasteurization
HP: high-temperature short-term pasteurization
