# Supplementary material for: Prevention of human milk-acquired cytomegalovirus infection in very-low-birth-weight infants
Source: BMC Pediatr. 2023 May 18;23:244. doi: 10.1186/s12887-023-04044-8 (PMC10193732; doi:10.1186/s12887-023-04044-8)
Supplement: Supplementary file 1 — Supplementary Material 1 [file 12887_2023_4044_MOESM1_ESM.docx]

**Supplemental material**

**Changes in the macronutrients of HM after preparation**

| **Variables** | **Change after treatment** | | | ***P-value****^1^* | **Post-hoc** |
| --- | --- | --- | --- | --- | --- |
|  | **FT** | **FT+LP** | **FT+HP** |  |  |
| **Energy (Kcal/dL)** | -2,04 ± 3.00 | -0.89 ± 8.64 | 0.41 ± 3.26 | *0.01* | FT < LP, HP |
| **Crude protein (g/dL)** | -0.05 ± 0.14 | 0.01 ± 0.35 | -0.01 ± 0.16 | *0.27* |  |
| **True Protein (g/dL)** | -0.06 ± 0.11 | 0.01 ± 0.30 | -0.01 ± 0.13 | *0.17* |  |
| **Fat (g/dL)** | -0.17 ± 0.31 | -0.07 ± 0.51 | 0.08 ± 0.59 | *0.13* |  |
| **Lactose (g/dL)** | 2.49 ± 12.25 | -0.08 ± 0.89 | 0.10 ± 0.40 | *0.33* |  |

Values are mean ± standard deviation.

^1^Wilcoxon’s signed-rank test

For post-hoc pairwise comparison for categorical variables, Scheffe’s or Dunn’s method was applied to adjust the significance level of alpha due to the multiple testing. Means with different scripts are different from each other (P<0.05).

**Abbreviations**: FT, freeze-thawing; LP, low-temperature Holder pasteurization; HP, high-temperature short-term pasteurization.
